# Cognitive and Behavioral Domains That Reliably Differentiate Normal Aging and Dementia in Down Syndrome

**DOI:** 10.3390/brainsci11091128

**Published:** 2021-08-25

**Authors:** Jordan P. Harp, Lisa M. Koehl, Kathryn L. Van Pelt, Christy L. Hom, Eric Doran, Elizabeth Head, Ira T. Lott, Frederick A. Schmitt

**Affiliations:** 1 Department of Neurology, University of Kentucky, Lexington, KY 40506, USA; lisa.mason@uky.edu; 2 Sanders-Brown Center on Aging, University of Kentucky, Lexington, KY 40506, USA; vanpelt.kathryn@gmail.com; 3 Department of Psychiatry and Human Behavior, University of California, Irvine, CA 92697, USA; homc@uci.edu; 4 Department of Pediatrics, School of Medicine, University of California, Irvine, CA 92697, USA; edoran@hs.uci.edu; 5 Department of Pathology & Laboratory Medicine, University of California, Irvine, CA 92697, USA; heade@uci.edu; 6 Departments of Neurology and Pediatrics, University of California, Irvine, CA 92697, USA; itlott@hs.uci.edu; 7 Department of Neurology and Sanders-Brown Center on Aging, University of Kentucky, Lexington, KY 40506, USA; fascom@uky.edu

**Keywords:** Down syndrome, dementia, cognition, functional independence, neuropsychological assessment, primary care, screening

## Abstract

Primary care integration of Down syndrome (DS)-specific dementia screening is strongly advised. The current study employed principal components analysis (PCA) and classification and regression tree (CART) analyses to identify an abbreviated battery for dementia classification. Scale- and subscale-level scores from 141 participants (no dementia *n* = 68; probable Alzheimer’s disease *n* = 73), for the Severe Impairment Battery (SIB), Dementia Scale for People with Learning Disabilities (DLD), and Vineland Adaptive Behavior Scales—Second Edition (Vineland-II) were analyzed. Two principle components (PC1, PC2) were identified with the odds of a probable dementia diagnosis increasing 2.54 times per PC1 unit increase and by 3.73 times per PC2 unit increase. CART analysis identified that the DLD sum of cognitive scores (SCS < 35 raw) and Vineland-II community subdomain (<36 raw) scores best classified dementia. No significant difference in the PCA versus CART area under the curve (AUC) was noted (D(65.196) = −0.57683; *p* = 0.57; PCA AUC = 0.87; CART AUC = 0.91). The PCA sensitivity was 80% and specificity was 70%; CART was 100% and specificity was 81%. These results support an abbreviated dementia screening battery to identify at-risk individuals with DS in primary care settings to guide specialized diagnostic referral.

## 1. Introduction

Down syndrome (DS), a genetic condition caused predominantly by the triplication of chromosome 21, is highly associated with the development of Alzheimer’s disease (AD) [1]. Chromosome 21 includes the amyloid precursor protein (APP) gene, and triplication results in overexpression of APP and related proteins, accelerating the accumulation of misfolded amyloid in the brain [2,3,4]. Additional AD risk factors are also associated with DS including a higher propensity for neuroinflammation, oxidative damage, sleep apnea, and reduced cognitive reserve due to premorbid intellectual disability [1,5,6,7]. Indeed, AD pathological changes have been documented in adults with DS as young as 20 years, and nearly all adults with DS show the amyloid plaques and neurofibrillary tangles associated with AD by 40 years of age [8,9,10].

DS is associated with different physical morphology, intellectual disabilities, and reduced lifespan compared to the typically developing population. Associated health problems include atlantoaxial instability, musculoskeletal and dental conditions, congenital heart disease, hematologic conditions, obesity, hypothyroidism, obstructive sleep apnea, impaired hearing and vision, and overall increased functional dependence due to behavioral, psychiatric, and intellectual impairments [7,11,12,13,14]. Advances in medical management of these co-morbidities have lowered mortality from early-life conditions, but one consequence of lengthened lifespan is that more individuals with DS now survive to the age of risk for AD [15].

Due to the need for preventative care and ongoing management of chronic health conditions associated with DS, health professionals and advocacy groups recommend the integration of DS-specific care in primary care settings [14,16,17]. Healthcare systems have made progress toward this end, but there is a need for improvement [11,18]. Cognitive screening and monitoring for dementia is particularly difficult, as cognitive measurement is complicated by pre-existing intellectual disability (ID), large inter- and intra-individual variability in cognition and behavior, tolerability of testing methods, and the lack of an identified “gold standard” neurocognitive battery, even for research purposes [19,20]. Moreover, neurocognitive tests are not feasible in primary care settings due to the lengthy procedures and specialized training needed for the interpretation of comprehensive evaluations.

In recent studies, our group has sought to establish an evidence base for abbreviated neurobehavioral examination procedures appropriate for in-office dementia monitoring by community practitioners caring for patients with DS [21]. Performance measures in our long-term cohort studies include the Brief Praxis Test (BPT) [22] and the Severe Impairment Battery (SIB) [23]. Informant measures included the Dementia Questionnaire for People with Learning Disabilities (DLD) [24] and Vineland Adaptive Behavior Scales-Second Edition (Vineland-II) [25]. The BPT, SIB, and DLD have all been used in early DS clinical trials assessing the effects of anticholinesterase therapy [26] as well as antioxidants [27,28]. Moreover, the SIB has long been validated as a cognitive measure for severe impaired individuals with AD [29]. The Vineland-II has been widely used and validated in the DS population [30,31,32] and adaptive behavior decline is a diagnostic criterion for AD, necessitating the inclusion of this type of measure in this study. These measures were selected at the outset of the two parent cohorts from which the present data are drawn, and target the domains of cognition (SIB, DLD), praxis (BPT), and functional independence (DLD, Vineland-II) that underlie both NINCDS-ADRDA and DSM-IV criteria for dementia/major neurocognitive disorder.

The present study seeks to further identify the key components that are useful for dementia detection through three aims:

- Aim 1: to identify the underlying components of a cognitive battery that was used to assess functioning in domains commonly affected by AD.
- Aim 2: to select the minimum necessary individual items or subscales using CART analysis to create an abbreviated battery for classifying AD status.
- Aim 3: to compare the classification accuracy between the two methods: components from the full battery vs. the abbreviated battery.

## 2. Materials and Methods

### 2.1. Description of Sample

The current study combines participants from cohorts at two different sites: the University of Kentucky and the University of California, Irvine (UCI). The University of Kentucky Aging and Down Syndrome (ADS) study is a longitudinal cohort of aging individuals with DS. For the purpose of the current study, only the baseline visit was used for 88 participants. Twenty-nine of the original one hundred and seventeen participants in the overall ADS study were unable to contribute data for the present analysis, predominantly due to inability to engage in testing because of advanced dementia. The University of Kentucky ADS cohort recruited individuals with DS between 25 and 64 years of age. From the UCI cohort, only the baseline visit was used for 53 participants. One of the original 54 participants in the overall UCI study was unable to contribute data for the present analysis due to inability to engage in testing because of advanced dementia. The UCI cohort included people between 43 and 58 years of age. 

A karyotype diagnosis of trisomy 21 (full or mosaic) or the Robertsonian translocation form of DS was required. Baseline levels of ID were determined by caregiver report of prior evaluation results or by a review of records when available. Other requirements for study inclusion included a stable medical condition for at least 3 months prior to the study and to have an absence of systemic disorders that might confound a diagnosis of dementia. Medication usage including psychotropic and Parkinsonian drugs was required to be stable for 3 months prior to study, and English-speaking skills were required to facilitate neuropsychological testing.

Research procedures were independently reviewed and approved by the University of Kentucky Institutional Review Board and the UCI Institutional Review Board. Participants completed approved protocols for informed consent or assent with guardian or legally authorized representative approval. 

### 2.2. Description of Measures 

Both sites administered a combination of performance and informant measures that have been used with adults with DS. Performance measures included the BPT and SIB, and informant measures included the DLD and Vineland-II. 

The BPT is a 20-item measure of dyspraxia that minimizes verbal demands in favor of simple behavioral output. Low scores on the BPT indicate severe dyspraxia. 

The SIB utilizes one-step commands and gestural cues, and allows for non-verbal responses and partially correct responses in order to assess cognition in individuals with severe dementia. The SIB yields a total score along with six major subscales for attention, orientation, language, memory, visuospatial ability, and construction, with additional scores for orientation to name, praxis, and social interaction. Lower scores indicate more severe deficits. 

The DLD is a 50-item informant questionnaire measuring behavioral and cognitive dysfunction. The DLD yields three scores: (1) sum of cognitive score (SCS), measuring short-term memory, long-term memory, and spatial/temporal orientation; (2) sum of social score (SOS), measuring speech, practical skills, mood, activity/interest, and behavioral disturbance; and (3) a total score that combines the SCS and SOS. DLD raters for the current study were caregivers and/or legal guardians responsible for the daily care of the participants either at home or an assisted living facility. Higher scores on the DLD indicate more severe impairment. 

The Vineland-II is an informant-based measure covering domains of communication, daily living skills, socialization, motor skills, and maladaptive behavior. The Vineland-II provides a composite score reflecting an individual’s overall adaptive behavior functioning, called the Adaptive Behavior Composite (ABC). The Vineland-II is administered by a trained interviewer to the parent or caregiver. 

#### 2.2.1. Consensus Diagnosis

AD diagnosis, based on NINCDS-ADRDA or DSM-IV criteria [33,34], was made at each site using a consensus process involving a neurologist and a psychologist. The SIB, BPT, and DLD test data were used in consensus diagnosis decisions. The diagnosis of dementia required a clinical and neurological examination showing deficits in 2 or more areas of cognitive functioning, and progressive worsening of cognitive performance compared to the potential participant’s baseline functioning.

#### 2.2.2. Data Preparation

Raw scores were used for all measures except for the Vineland-II domain-level scores (ABC, communication, daily living skills, socialization, and motor skills), which were only available as standardized scores. No subscales were removed for excessive missingness (>15% of data points missing across individuals). The DLD had the least amount of missing data (0.71% missing), followed by the Vineland-II (9.22% missing) and the SIB (9.93% missing). Missing data were imputed using chained random forests via the ‘missRanger’ R package [35]. Next, the DLD scores were inverted for consistent directionality with the other measures. All analyses were completed in R v 4.0.0 [36] and the significance level set to 0.05.

Aim 1: Principal Components Analysis

For the first aim, principal components analysis (PCA) was used to identify the number of components assessed by all individual items from the performance and informant procedures. The R package ‘tidymodels’ was used for all steps of the PCA analysis. The appropriateness of using PCA was evaluated using variable correlations, Bartlett’s test, Kaiser-Meyer-Olkin, and determinants. In terms of correlations, variables should be only mildly intercorrelated and were examined using thresholds suggested by Field et al. [37] to have absolute correlations ranging from 0.3 to 0.9. Items with more than one occurrence for a correlation outside of the range were excluded from the PCA analysis. Only four variables needed to be excluded: Vineland-II ABC, Vineland-II social domain score, one item from the Vineland-II maladaptive behavior domain scale, and the SIB total score. The Kaiser-Meyer-Olkin factor adequacy was 0.95, above the 0.7 threshold. Bartlett’s test of sphericity was significant (X^2^(325) = 5207.62; *p* < 0.001). Finally, the determinant was below 0.00001. Together, these indicated that PCA was appropriate. Based on the scree plot of unrotated results, two components with eigenvalues > 1.0 were identified, accounting for 76% of the total variance in scores. Varimax rotation of loadings was then employed to enhance interpretability of identified components.

The dataset containing the two components scores and AD diagnostic status were then split into a training and test dataset. The training dataset was used to generate the logistic regression model. The model was assessed for multicollinearity and the assumption that independent variables are linearly related to the log odds. The performance of the generated model was assessed on the test dataset by evaluating the area under the curve (AUC), sensitivity, and specificity. 

Aim 2: Classification and Regression Tree Analysis

For the second aim, classification and regression tree (CART) modeling was used to identify an optimal set of rules for classifying participants by diagnosis based only on item- and subscale-level data from the neurobehavioral battery. Again, R package ‘tidymodels’ was used in all steps of the CART analysis. First, training and test datasets were generated from the data. Then, the training dataset was used to generate a set of 10-fold cross-validation samples for model hyperparameter tuning. The best hyperparameters were selected based on the AUC. The CART model was first fit on the training dataset, then on the test dataset to assess performance. 

Aim 3: Receiver Operating Characteristic Curves and Comparisons

For the third aim, to compare the relative utility of the PCA and CART models, the AUC of both models for classifying diagnosis were compared using the bootstrap test for comparing ROC curves (R routine ‘roc.test’ from the package ‘pROC’). Sensitivity, specificity, and positive and negative predictive values were computed for the PCA and CART models.

## 3. Results

### 3.1. Sample Characteristics

A total of 141 participants were included in the current study. Just over half of the participants (*n* = 73; 51.77%) were diagnosed with probable AD. Full participant characteristics are provided in Table 1.

### 3.2. Aim 1: Principal Components Analysis Results

Results of the PCA are listed in Supplementary Table S1. The two components could not be easily labeled because they each contained items from communication, daily living skills, and cognitive domains. For the PCA method, logistic regression results demonstrated that higher scores on PC1 and PC2 were predictive of AD diagnosis. For each unit increase in PC1, the odds of a probable dementia diagnosis increased 2.54 times, and for each unit increase in PC2 the odds of a probable dementia diagnosis increased 3.73 times (Table 2).

### 3.3. Aim 2: Classification and Regression Tree Analysis Results 

**Figure 1:**
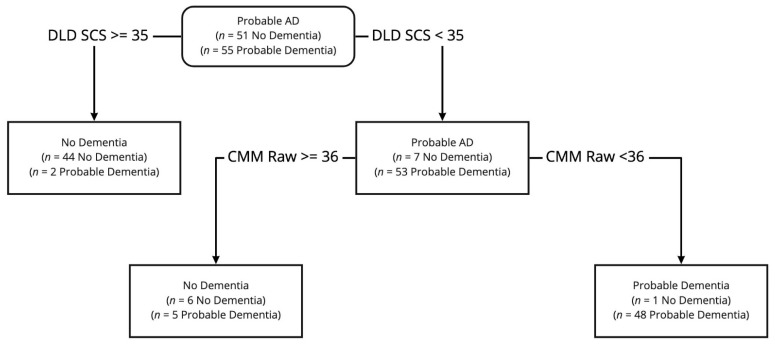
Results of CART analysis. *Note.* AD = Alzheimer’s disease; DLD SCS = Dementia Questionnaire for People with Learning Disabilities (DLD) sum of cognitive scores raw score; CMM Raw = Vineland-II community subdomain raw score.

The CART analysis revealed that the DLD SCS and Vineland-II community subdomain raw scores best-classified dementia (Figure 1). A DLD SCS less than 35 and a Vineland community score less than 34 are indicative of AD dementia.

### 3.4. Aim 3: Comparison of PCA and CART Model Classification Utility

**Figure 2:**
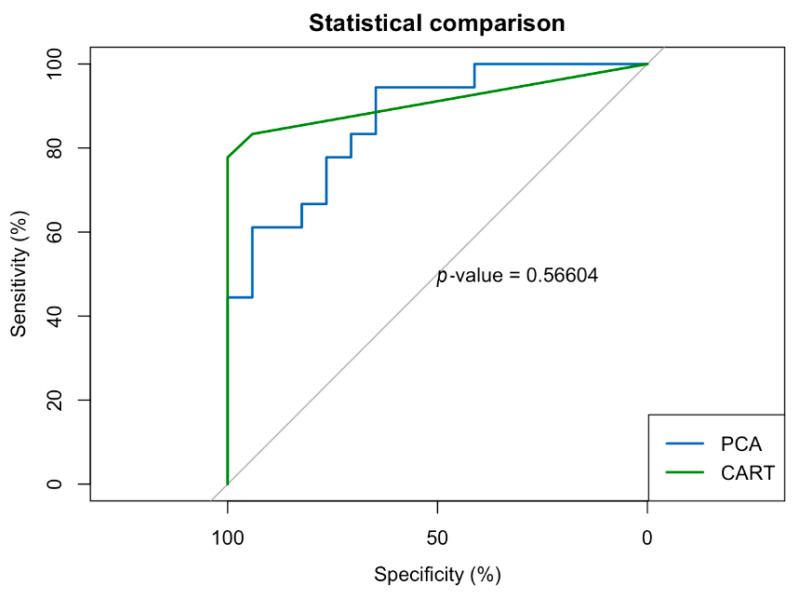
ROC curve comparison for PCA versus CART derived models. PCA area under the curve (AUC) = 0.87, and CART model AUC = 0.91.

Comparing the PCA logistic regression and CART classification methods, there was no significant difference in AUC (D(65.196) = −0.57683; *p* = 0.57) (Figure 2). The PCA analysis resulted in an AUC of 0.87 while the CART model produced an AUC of 0.91. In terms of classification utility, the PCA model showed very good sensitivity (0.80) and good specificity (0.70), with high negative predictive value (0.824) and moderately high positive predictive value (0.667) at the combined sample base rate. The CART model demonstrated excellent sensitivity (1.00) and very good specificity (0.810), with excellent negative predictive value (1.00) and high positive predictive value (0.778) at the combined sample base rate.

## 4. Discussion

The present data indicate that for adults with DS, variability in orientation, language, memory, visuospatial skills, praxis, mood, and social participation is largely explained by two underlying principal components. These two components seemed to differentiate cognitive from practical function (i.e., the ability to answer questions vs. the ability to carry out everyday tasks). Additionally, the use of the two-component model to categorize participants with respect to AD dementia status showed high classification accuracy. These findings support the further distillation of the modest-sized battery into a “short form” that can be easily administered in a primary care setting. Additionally, it takes less than an hour to administer to an informant who knows the patient with DS well, and requires minimal office space and test stimuli.

Results of the CART analysis also demonstrated that a small subset of the original battery—the cognitive subscale of the DLD (SCS) and the community subscale of the Vineland-II—were just as effective in classifying AD dementia status. However, the CART model exhibited better negative predictive value, in that fewer participants with dementia were misclassified as non-demented compared to the principal components model. A short battery based on the CART model is also quicker to administer and can in most cases be completed in less than 30 min.

A key finding is that the two contributory measures are not direct, objective measures of cognitive performance completed by the patient. Instead, they are informant-based scores of the patient’s observed changes in cognitive abilities (DLD-SCS) and self-management in community tasks (Vineland-II community). Unexpectedly, classification did not appreciably hinge on objective, performance-based neurocognitive measures. This highlights the critical component of informed caregiver ratings when screening for dementia in DS populations and provides some assurance that differential diagnosis of AD dementia is still possible when a patient’s cognitive abilities cannot be directly assessed due to profound ID, limited cooperation, sensory impairments, or speech and language disorders.

Overall, the present data suggest that in clinical contexts with limited time and access to advanced training in test administration, the cognitive subscale of the DLD and community subscale of the Vineland-II, two widely available instruments, may suffice for screening and monitoring purposes. To be clear, we do not conclude that these two subscales constitute a comprehensive research or diagnostic battery, as definitive diagnosis should be based on longitudinal data. Nor is it the case that objective neurocognitive performance measures are redundant for diagnostic purposes. On the contrary, diagnostic criteria require objective neurocognitive assessment in order to make a firm diagnosis [33]. The present analysis was conducted for the specific aims of the study, namely identifying measures for resource-limited healthcare settings to encourage wide adoption of dementia screening among community DS practitioners. Prior efforts to use data reduction approaches to streamline a cognitive and behavioral battery for dementia in DS were focused primarily on developing a minimal comprehensive battery for research and specialty evaluation settings; thus, the resulting recommendations were not as relevant to primary care screening [38].

Furthermore, the present findings do not suggest that these two subscales represent an advancement in the early detection of AD dementia relative to more comprehensive test batteries. Instead, the benefit of adopting a minimal screening battery would enable more of the broader DS population to be evaluated, who may otherwise go unassessed. At the individual level, “early” detection is relative to the person’s typical access to care, not the recommended standard of care. Given that nearly half of adults with DS do not receive regular screening for typical DS-associated health problems [11], it is reasonable to cast a wider net with “good enough” measures easily administered in primary care settings. Moreover, operating characteristics of the CART model align with a preference for high sensitivity (potential over-identification) over high specificity because the goal of screening is to provide support to this population.

Examination of the factor structure and of the item- and subscale-level operating characteristics in diagnostic batteries for dementia in DS is relatively new ground, and it is difficult to contextualize the present findings in the literature on the constructs measured. Broadly speaking, these data are in line with indications that adults with DS have reduced—but not absent—functional independence relative to other adults with intellectual disabilities [39], and dementia-related impairment in that domain may be captured by a community functioning measure such as the Vineland-II community subscale. Prior work using the Vineland-II to predict AD dementia in DS found that informant-rated receptive language skills, in addition to performance on a semantic verbal fluency task, were strong indicators of mild cognitive impairment in DS [40]. The present analysis instead examined individuals with and without AD dementia and found community management skills to be the most informative subscale of the Vineland-II. These findings are not contradictory, as in the present study it is likely that variability between participants cognitive functioning were captured by the DLD-SCS informant-based score, leaving more contextual community-based functioning to be best represented by the Vineland-II community subscale.

Beyond those discussed above, additional limitations of this study include the use of the SIB, DLD, and BPT along with the neurologic examination to determine consensus diagnosis. Our prior investigations have found that in 96% of cases, the final consensus diagnosis matched the neurologist’s diagnosis that was formed independently of the SIB, BPT, and DLD scores. Still, discussion with the informant allows exposure to much of the same information captured by these instruments, and consideration of this information when forming a diagnosis is unavoidable. The eventual goal of both study cohorts is to substantiate consensus diagnoses with neuropathology at autopsy, allowing a more direct evaluation of the influence of potential criterion contamination. Additionally, the present study relied on informants who were very familiar with the participants with DS being rated, and in many cases, such a source of information cannot be found in practice.

## Figures and Tables

**Table 1:** Participant Characteristics.

Characteristic	No Dementia, *N* = 68	Probable AD, *N* = 73	Overall, *N* = 141
Sex			
Female	36 (52.94%)	41 (56.16%)	77 (54.61%)
Male	32 (47.06%)	32 (43.84%)	64 (45.39%)
Age (years)	38.11 (9.34)	52.68 (6.12)	45.66 (10.70)
Level of Intellectual Disability (estimated)			
Mild	3 (4.41%)	14 (19.18%)	17 (12.06%)
Moderate	36 (52.94%)	29 (39.73%)	65 (46.10%)
Profound	28 (41.18%)	15 (20.55%)	43 (30.50%)
Severe	1 (1.47%)	13 (17.81%)	14 (9.93%)
Unknown	0 (0.00%)	2 (2.74%)	2 (1.42%)
Site			
UCI	0 (0.00%)	53 (72.60%)	53 (37.59%)
UKY	68 (100.00%)	20 (27.40%)	88 (62.41%)

*n* (%); mean (SD).

**Table 2:** Logistic Regression.

Predictors	Odds Ratio	95% *CI*	*p*
(Intercept)	1.14	0.46–2.84	0.773
PC1	2.54	1.69–3.81	<0.001
PC2	3.73	1.62–8.60	0.002
Observations	106		
Tjur’s R^2^	0.786		

*n* (%); mean (SD).

## Data Availability

The data presented in this study are openly available in OSFHome at [DOI 10.17605/OSF.IO/EK3YH].

## Supplementary Materials

The following are available online at https://www.mdpi.com/article/10.3390/brainsci11091128/s1, Table S1: Principal Components Analysis Results.
