# Supplementary material for: Cognitive and Behavioral Domains That Reliably Differentiate Normal Aging and Dementia in Down Syndrome
Source: Brain Sci. 2021 Aug 25;11(9):1128. doi: 10.3390/brainsci11091128 (PMC8468129; doi:10.3390/brainsci11091128)
Supplement: Supplementary file 1 [file brainsci-11-01128-s001.zip › brainsci-1322035-supplementary.pdf]

Table S1. Principal Components Analysis Results

Scale/Subscale	PC1 Loading	PC2 Loading	Highest Loading
SIB Orientation	-0.190	0.158	PC1
SIB Construction	-0.198	0.151	PC1
Vineland-II Communication—Receptive	-0.212	-0.104	PC1
Vineland-II Communication—Expressive	-0.216	-0.154	PC1
Vineland-II Daily Living Skills—Personal	-0.200	0.116	PC1
Vineland-II Daily Living Skills—Domestic	-0.213	-0.144	PC1
Vineland-II Daily Living Skills—Community	-0.215	-0.173	PC1
Vineland-II Socialization—Interpersonal Relationships	-0.217	-0.142	PC1
Vineland-II Socialization—Play and Leisure	-0.208	-0.199	PC1
Vineland-II Socialization—Coping Skills	-0.211	-0.204	PC1
Vineland-II Motor Skills—Gross	-0.207	-0.180	PC1
Vineland-II Motor Skills—Fine	-0.214	-0.171	PC1
Vineland-II Motor Skills Domain	-0.188	-0.030	PC1
DLD Sum of Cognitive	-0.216	-0.010	PC1
DLD Sum of Social	-0.199	0.093	PC1
Brief Praxis Test	-0.200	0.280	PC2
SIB Social Interaction	-0.129	0.279	PC2
SIB Memory	-0.194	0.209	PC2
SIB Orientation to Name	-0.124	0.227	PC2
SIB Attention	-0.194	0.213	PC2
SIB Praxis	-0.124	0.332	PC2
SIB Language	-0.211	0.227	PC2
SIB Visuospatial	-0.197	0.232	PC2
Vineland-II Communication—Written	-0.204	-0.235	PC2
Vineland-II Communication Domain	-0.150	-0.271	PC2
Vineland-II Daily Living Skills Domain	-0.187	-0.200	PC2

Note: SIB: Severe Impairment Battery; DLD: Dementia Questionnaire for People with Learning Disabilities; Vineland-II: Vineland Adaptive Behavior Scales, Second Edition; PC: Principal Component
